# A Low-loss Metasurface Antireflection Coating on Dispersive Surface Plasmon Structure

**DOI:** 10.1038/srep36190

**Published:** 2016-11-02

**Authors:** Jiyeon Jeon, Khagendra Bhattarai, Deok-Kee Kim, Jun Oh Kim, Augustine Urbas, Sang Jun Lee, Zahyun Ku, Jiangfeng Zhou

**Affiliations:** 1 Division of Convergence Technology, Korea Research Institute of Standards and Science, Daejeon, 305-340, Korea; 2 Department of Electrical Engineering, Sejong University, Seoul 143-747, Korea; 3 Department of Physics, University of South Florida, Tampa, 33620, USA; 4 Air Force Research Laboratory, Materials Directorate, Wright-Patterson AFB, 45433, USA

## Abstract

Over the years, there has been increasing interest in the integration of metal hole array (MHA) with optoelectronic devices, as a result of enhanced coupling of incident light into the active layer of devices via surface plasmon polariton (SPP) resonances. However, not all incident light contributes to the SPP resonances due to significant reflection loss at the interface between incident medium and MHA. Conventional thin-film antireflection (AR) coating typically does not work well due to non-existing material satisfying the AR condition with strong dispersion of MHA’s effective impedances. We demonstrate a single-layer metasurface AR coating that completely eliminates the refection and significantly increases the transmission at the SPP resonances. Operating at off-resonance wavelengths, the metasurface exhibits extremely low loss and does not show resonant coupling with the MHA layer. The SPP resonance wavelengths of MHA layer are unaffected whereas the surface wave is significantly increased, thereby paving the way for improved performance of optoelectronic devices. With an improved retrieval method, the metasurface is proved to exhibit a high effective permittivity (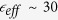) and extremely low loss (tan *δ* ~ 0.005). A classical thin-film AR coating mechanism is identified through analytical derivations and numerical simulations.

When electromagnetic waves encounter the interface between two media with different refractive indices, the energy of incident light is partially reflected while the rest propagates into the second medium. The undesired reflection can severely limit the performance of modern optoelectronic devices, such as photovoltaic cells, light-emitting diodes, and infrared detectors, etc. Extensive efforts have been made to develop antireflection (AR) techniques to reduce the amount of reflective losses. Conventionally, a layer of quarter-wavelength-thick dielectric coating has been used to suppress the reflection at certain wavelength owing to the destructive interference. To completely eliminate the reflection, the dielectric coating has to satisfy the AR condition, 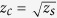 and *t*_*c*_ = *λ*/4, where *t*_*c*_ is the thickness of the coating layer, *λ* is the wavelength in the coating material, 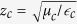
and 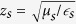 are the impedances, 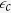 and 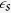 are permittivities, and *μ*_*c*_ and *μ*_*s*_ are permeabilities of the coating material and substrate, respectively. However, due to the lack of coating material with accurate impedance at wavelengths of practical applications, a perfect elimination of reflection is usually unachievable. Metamaterials (MMs) are artificially structured materials that provide the tunable effective permittivity and effective permeability by varying their geometric design, thereby enabling to achieve the required AR impedance matching conditions. Recent progress in MMs has demonstrated AR coating on non-dispersive semiconductor surfaces^1^,^2^,^3^. It has been shown that a metallic-resonator/dielectric/metallic-mesh sandwich-type MM can be used as AR coating on a gallium arsenide (GaAs) substrate^1^. In this work, destructive interference of light reflected by two metallic structure layers eliminates the overall reflection within certain wavelength range in the THz regime. A more recent work has demonstrated that an array of metallic nanoantenna buried between an amorphous silicon (α-Si) film and crystalline silicon substrate can effectively reduce the reflection between air and silicon substrate^2^. An array of metallic cross-wires on top of a low refractive index magnesium fluoride (MgF_2_) dielectric film can also dramatically reduce the reflection from germanium (Ge) substrate^3^. In these reports, MM based AR coatings were applied to homogenous material (GaAs, Si or Ge) with nearly constant impedance. However, it has not been clarified to date whether MM based AR coating can be applicable to the resonant metallic structures fabricated on substrate and by extension, integrated with optoelectronic devices^4^,^5^,^6^,^7^. MMs are typically made from metallic resonators. Accordingly, MM based AR coating may cause the AR impedance matching to malfunction due to unwanted resonance coupling^8^ with metallic structures underneath. In addition, the expected functionality resulting from metallic structures on substrate or devices (e.g. extraordinary optical transmission (EOT), improved device performance) may be affected.

One example of metallic structures integrated with substrate or optoelectronic devices^4^,^5^,^6^,^7^ is a metallic hole array (MHA) which exhibits strong surface plasmon polarition (SPP) resonances. SPPs are the collective oscillations of electron plasma in the metallic structure excited by electromagnetic (EM) radiation^9^. The SPP resonances occurring on various types of periodic arrays of subwavelength holes in the metallic film lead to EOT^10^. At the resonance wavelengths, SPPs help to concentrate light into subwavelength scale beyond the diffraction limit and also assist in significantly enhancing the EM field. These characteristic features have been utilized in many applications including surface-enhanced Raman spectroscopy^11^, bio- or chemical-sensors^12^, photonic circuits^13^,^14^ and photovoltaic devices^15^. In particular, for MHA structure on a dielectric substrate, when the wave vector of normally incident light matches the reciprocal lattice of the array, strong SPP resonances occur and produce evanescent waves that tunnel through subwavelength apertures, resulting in extraordinary transmission of light on the other side^10^,^16^. However, not all incident light contribute to the SPP resonances due to the impedance mismatch at the air-MHA interface (i.e. a part of incident light can be reflected). It has been recently demonstrated that a thin dielectric film used as an AR coating can effectively improve the transmission through MHA at the resonant wavelengths^17^. Another recent study shows that with proper thin dielectric film AR coating, a gold (Au) MHA’s grating layer can effectively enhance the absorption of light in the underneath active quantum well device region by 13 times^18^. However, the resonance wavelength is tunable with MHA geometry (e.g. periodicity or aperture size), so it is impossible to find a common material with appropriate impedance that matches the AR condition,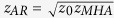, for different wavelengths in various applications. Furthermore, the effective impedance of MHA, *z*_*MHA*_, exhibits strong dispersion around the SPP resonance wavelength. As previously stated, MM based AR coating can provide great flexibility to solve these challenges, but the resonance of MM may be able to generate the interaction with the SPP resonance of MHA, leading to the shift of SPP resonance wavelength and damping or degradation of surface wave^8^.

In this paper, we demonstrate an off-resonance, disk-type MM operating as the AR (Meta-AR) coating for a MHA layer fabricated on a GaAs substrate. The Meta-AR coating can effectively reduce the reflection at both first-order and second-order SPP resonances, thereby increasing the transmission. Additionally, our results do not show the resonance shift or damping of the SPP because MM does not couple with SPP resonance. Instead, the surface wave is enhanced as compared to the uncoated MHA (no AR coating atop MHA). Furthermore, we demonstrate that MM layer works as a homogenous thin film with high dielectric constant (~30) and extremely low loss (loss tangent ~0.005). Thus the Meta-AR coating can be understood as well-known thin-film AR coating, which provides an intuitive purely optical model compared to the electrical transmission-line model used in previous work^2^. We also validate through simulation that such thin-film model can be generally applicable to other MM-based AR coating structures^1^,^2^.

## Results

### Metasurface antireflection coating

**Figure 1:**
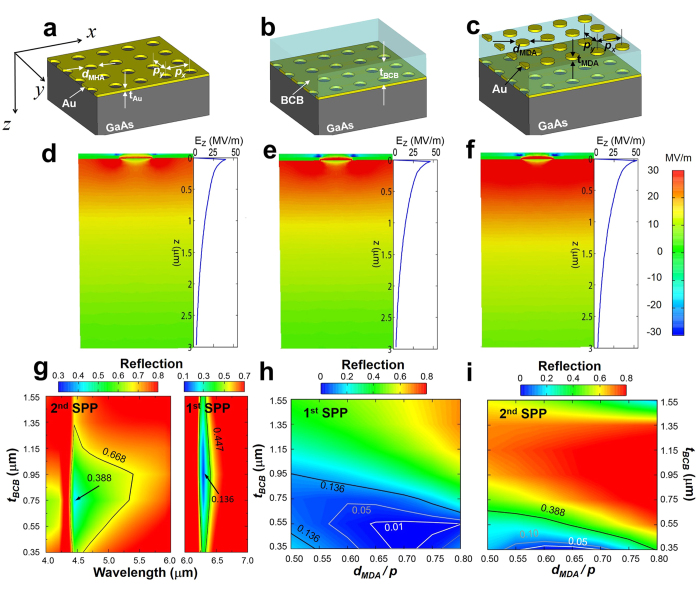
Illustrations of (**a**) MHA, (**b**) BCB layer coated MHA and (**c**) MHA coated with MDA atop the BCB layer. (**d**–**f**) *E*_*z*_ distribution at the first-order SPP resonance *λ*_1_ = 6.25 μm in *x* = 0.36 μm plane of a unit cell (*x* = 0 at the center of the unit cell) for structures shown in (**a**–**c**), respectively. The interface between MHA and GaAs substrate is set to zero in *z*-axis (*z* = 0). (**g**) Simulated reflection colormap for structure displayed in (**b**) as a function of wavelength and the BCB thickness *t*_*BCB*_. Colormaps of simulated reflection at (**h**) the first-order and (**i**) the second-order SPP resonance wavelengths for structure as shown in (**c**) as a function of BCB thickness *t*_*BCB*_ and the ratio *d*_*MDA*_/*p*.

Our metamaterial based antireflection (Meta-AR) coating consists of a planar metallic disk array (MDA) on the top of a cured layer of benzencyclobutene (BCB). For comparison of Meta-AR coating with conventional AR coating (a BCB layer only), we designed three types of samples, namely the uncoated MHA, a BCB layer coated MHA and MHA coated with an array of circular Au disks (MDA) atop the BCB layer. Figure 1a–c illustrate three structures on GaAs substrates, respectively. In all structures, the orthogonal pitches of MHA, P_*x*_ (pitch along *x*-axis) and *p*_*y*_ (pitch along *y*-axis), are both fixed at 1.8 μm (*p*_*x*_ = *p*_*y*_ = *p*) and the diameter of the circular aperture (*d*_*MHA*_) and thickness of Au (*t*_*Au*_) are 0.9 μm (a half of pitch, 0.5∙*p*) and 0.05 μm, respectively. We performed numerical simulations using CST Microwave Studio^19^, which utilizes a finite integration technique to obtain the solutions of Maxwell’s equations. In our simulation, the dielectric constant for GaAs and BCB are *ε*_*GaAs*_ = 11.56 and *ε*_*BCB*_ = 2.37, respectively. Au is described by a Drude model with plasma frequency, *ω*_*p*_ = 9.02 eV and collision frequency, *ω*_*c*_ = 0.038 eV^20^. Figure 1d–f show the simulated z component-electric field (*E*_*z*_) distribution in a plane parallel to *yz-*plane through the center of the unit cell (the distance between the parallel planes is 0.36 μm) when *x*-polarized light is incident along the normal direction. The surface waves are observed in all three structures, evidenced by the exponential decay of *E*_*z*_ along *z*-direction. This indicates that the SPP waves are confined to MHA-GaAs interface and propagate along the lateral direction. As compared with uncoated MHA (Fig. 1d: *p* = 1.8 μm, *d*_*MHA*_ = 0.9 μm), *E*_*z*_ of the SPP waves for BCB coated MHA (Fig. 1e: *t*_*BCB*_ = 0.95 μm) and Meta-AR coated MHA (Fig. 1f: *t*_*BCB*_ = 0.5 μm, *p* = 1.8 μm, *d*_*MDA*_ = 1.4 μm) are enhanced by ~20% and ~33%, respectively. The intensity of surface wave, *I* ∝ |*E*_*z*_|^2^, are remarkably increased by ~44% and ~77%, respectively. Without AR coating, the reflection of MHA at the first-order (*λ*_1_ = 6.25 μm) and the second-order (*λ*_2_ = 4.38 μm) SPP resonances are *R*_1_ ≈ 0.45 and *R*_2_ ≈ 0.67, respectively. This indicates that ~45% and ~67% of incident light do not contribute to the surface waves at the two lowest-order SPP resonances.

Figure 1g shows the simulated reflection of a BCB layer coated MHA sample (Fig. 1b) with thickness *t*_*BCB*_ varying from 0.35 μm to 1.55 μm. In the region enclosed by black curves, the reflection is reduced as compared with the uncoated MHA. However, reflection cannot be completely eliminated with only BCB coating because the impedance of the BCB (*Z*_*BCB*_ = 1/*n*_*BCB*_) does not match the AR condition (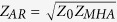) at these resonance wavelengths. The minimum values of reflection for the first-order and the second-order SPP resonances, *min*(*R*_1_) and *min*(*R*_2_) reach 0.136 and 0.388 with *t*_*BCB*_ = 0.95 μm and 0.75 μm, respectively. These optimal thicknesses can be well explained by the destructive interference of light reflected at the top (air-BCB interface) and bottom (BCB-MHA interface) surfaces of BCB layer. At the resonance wavelengths, the impedance of BCB layer is smaller than the impedance of air but larger than the effective impedance of MHA structure^17^, i.e. *Z*_*air*_ (=1) > *Z*_*BCB*_ > *Z*_*MHA*_. So both the lights reflected at the air-BCB and the BCB-MHA interfaces exhibit a phase shift of *π* with respect to the corresponding incident light at each interface. This leads to the condition for destructive interference, *t*_*BCB*_ = *λ*/4*n*_*BCB*_ = ~1.02 μm and ~0.71 μm, at the first and the second-order SPP resonances, respectively. With a properly designed MDA on the top of the BCB layer, the reflection can be further reduced and reach nearly zero at SPP resonance wavelengths. Figure 1h,i show the reflections taken at the SPP resonance wavelengths (*λ*_1_ and *λ*_2_) for the MHA structures with a MDA layer added on top of the BCB layer. In order to investigate the geometry dependence, *t*_*BCB*_ and *d*_*MDA*_ are varied from 0.35 μm to 1.55 μm and from 0.5∙*p* to 0.8∙*p*, respectively. The contour lines in black show the minimum reflection of only BCB layer coated MHA (*min (R*_1_) = 0.136 and *min* (R_2_) = 0.388). In between these lines shows a large region that the Meta-AR coating outperforms the BCB coating. For the first SPP resonance (Fig. 1h), the white contour line shows a wide range of 0.35 μm < *t*_*BCB*_ < 0.55 μm and 0.65 · *p* < *d*_*MDA*_ < 0.8 · *p* that reduces the reflection below 0.01 (*R*_1_ < 0.01). This shows that our Meta-AR (MDA atop BCB layer) coating is robust against possible fabrication tolerance, i.e. variations of BCB thickness (*t*_*BCB*_) and MDA size (*d*_*MDA*_). Reflections reach the minimum values, *min*(*R*_1_) = 1.21 × 10^−4^ and *min*(*R*_2_) = 3.16 × 10^−3^ when *t*_*BCB*_ = 0.55 μm and *d*_*MDA*_ = 0.75 · *p* and *t*_*BCB*_ = 0.35 μm and *d*_*MDA*_ = 0.65 · *p* at two resonance wavelengths, respectively. Two regions of minimum values of *R*_1_ and *R*_2_ do not overlap perfectly. For this reason, we mainly focus on AR coating designs that eliminate the reflection at the first-order SPP resonance (*R*_1_), which can also substantially reduce the reflection at the second-order SPP resonance (*R*_2_). Based on the simulation results, three type of structures illustrated in Fig. 1a–c have been fabricated.

**Figure 2:**
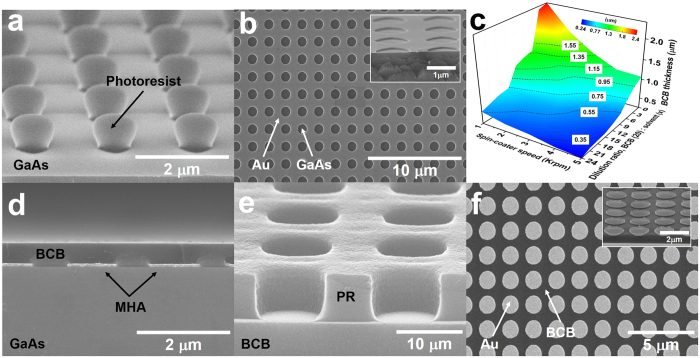
Scanning electron microscope (SEM) images of three samples (MHA, a BCB layer coated MHA, MHA coated with an array of circular metal disks atop the BCB layer) and BCB coating condition. (**a**) A periodic circular post photoresist (PR) pattern defined by standard photolithography. (**b**) E-beam deposition (5 nm of Ti and 50 nm of Au in sequence) and a liftoff processing, which leads to MHA structure. (**c**) Measured BCB thickness as a function of spin-coating speed and dilution ratio between BCB and rinse solvent. (**d**) BCB coated on MHA sample showing the flat-top surface. (**e**) A periodic circular hole PR pattern on the BCB layer shown in (**d**). Consecutive e-beam evaporations were used to deposit Ti (5 nm)/Au (50 nm) after (**e**), followed by a lift-off step. (**f**) Completed Meta-AR coated MHA (an array of circular metal disks atop the BCB coated MHA). Insets display the magnified MHA and MDA in Meta-AR coating.

The structure of all samples discussed here consists of a semi-insulating double-polished GaAs (100) substrate with MHA, BCB layer coated MHA, or Meta-AR (MDA atop BCB layer) coated MHA. In brief, the processing steps to fabricate the aforementioned samples are as follows. (i) Conventional photolithography was used to produce periodic circular post arrays in the photoresist (PR) layer (Fig. 2a). (ii) 5-nm-thick adhesion layer of titanium and 50-nm-thick layer of Au were deposited using e-beam evaporation and a liftoff processing, resulting in MHA structure (Fig. 2b). (iii) Based on the colormap of measured BCB film thickness (Fig. 2c) depending on spin-coating speed and dilution ratio between BCB and rinse solvent (Cyclotene 3022-35 and T1100, The Dow Chemical Company), BCB was spin-coated on MHA, whose top-surface is flat and smooth (Fig. 2d). (iv) A periodic circular hole pattern in the PR layer was defined by photolithography once again (Fig. 2e), followed by e-beam deposition of a 50-nm-thick layer of Au. (v) After the lift-off processing to remove the PR layer, the Meta-AR coated MHA was finally obtained as shown in Fig. 2f. The detailed fabrication is included in Supporting Information.

### Transmission enhancement due to Meta-AR coating

**Figure 3:**
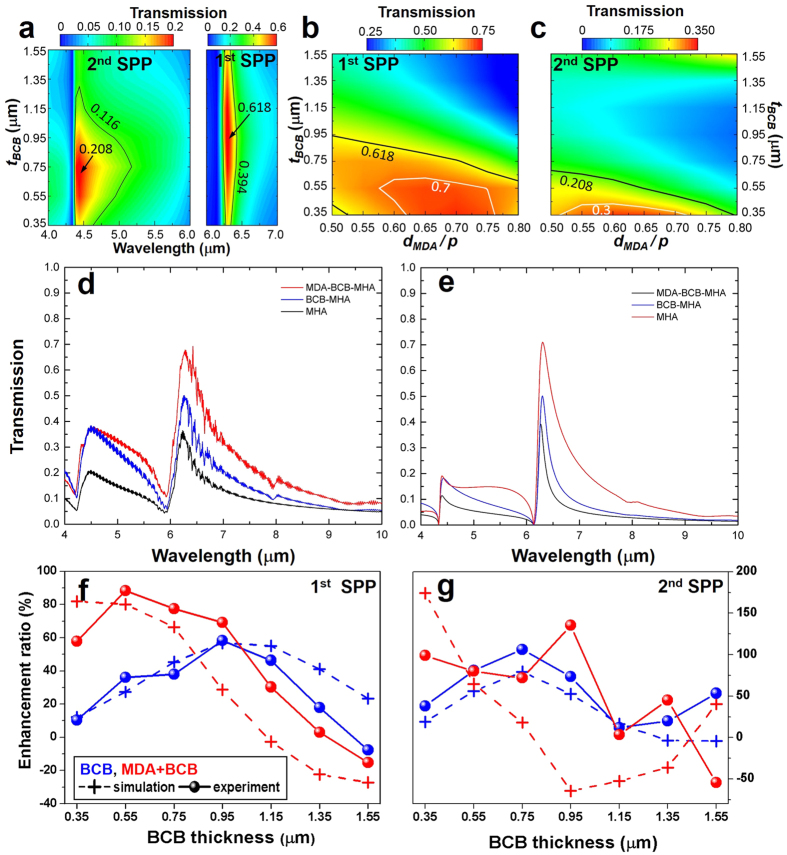
(**a**) Colormap of simulated transmission for BCB layer coated MHA as *t*_*BCB*_ increases from 0.35 μm to 1.55 μm with 0.2 μm step. Simulated transmission for MHA with Meta-AR coating at (**b**) the first-order and (**c**) second-order SPP resonance wavelengths when *d*_*MDA*_ is varied from 0.9 μm (0.5 · *p*) to 1.44 μm (0.8 · *p*) with a step of 0.09 μm (0.05 · *p*) and *t*_*BCB*_ is changed in the same manner as (**a**). (**d**) Measured and (**e**) simulated transmission for MHA, MHA with BCB coating and MHA with MDA+BCB coating when *t*_*BCB*_ = 0.55 *μm*. Experimental (sphere) and simulated (cross) transmission enhancement ratio for BCB (blue) and Meta-AR (red) coating at (**f**) the first-order and (**g**) second-order SPP resonance wavelengths.

As discussed in Fig. 1, the Meta-AR coating can effectively reduce the reflection at SPP resonance wavelengths, which leads to significant increase of transmission. The uncoated MHA (no AR coating atop MHA) exhibits low transmission where *T*_1_ ≈ 0.39 and *T*_2_ ≈ 0.12, at the first-order and second-order SPP resonances, respectively. Figure 3a–c show the simulated transmission of MHA with the BCB coating (Fig. 3a) and Meta-AR coating (Fig. 3b,c) as *t*_*BCB*_ and *d*_*MDA*_ vary within the range of 0.35 μm ≤ *t*_*BCB*_ ≤ 1.55 μm and 0.5 · *p* ≤ *d*_*MDA*_ ≤ 0.8 · *p*. Note that the maximum values of transmission are achieved with identical *t*_*BCB*_ and *d*_*MDA*_ to the minimum values of reflection as shown in Fig. 1g–i. Specifically, the regions enclosed by the black contour lines in Fig. 3a indicate the enhanced transmission due to BCB coating. Within these regions, transmission reaches the maximum values, *T*_1_ = 0.618 when *t*_*BCB*_ = 0.95 μm and *T*_2_ = 0.208 when *t*_*BCB*_ = 0.75 μm at the first-order and second-order SPP resonances, respectively. Owing to additional MDA design on top of the BCB layer, the transmission can further be improved up to *T*_1_ = 0.717 and *T*_2_ = 0.318 when *t*_*BCB*_ = 0.35 μm and *d*_*MDA*_ = 0.7 · *p*, as shown in Fig. 3b,c. The regions where the Meta-AR coating (MDA-BCB coating) outperforms the BCB coating are enclosed by black contour lines (Fig. 3b,c). The measured transmission spectra (Fig. 3d) show excellent agreement with simulations (Fig. 3e). In Fig. 3d, we also observe that the MDA+BCB coating significantly increases the transmission at SPP resonances. Figure 3f,g show the measured and simulated enhancement ratio (ER) of transmission for seven samples with various thickness of BCB layer (*t*_*BCB*_ is varied from 0.35 μm to 1.55 μm with a step of 0.2 μm), however pitch *p* and disk size *d*_*MDA*_ are fixed at *p* = 1.8 μm and 1.26 μm (0.7∙*p*), respectively. Here the ER is calculated by *ER* = (*T*_*i*_ − *T*_*MHA*_)/*T*_*MHA*_, where *i*’s are the BCB and Meta-AR (MDA+BCB). The overall agreement between experimental (solid lines) and simulated (dashed lines) ERs at the first-order SPP resonance is apparent from Fig. 3f. We find the maxima of measured ERs for BCB and Meta-AR coating are obtained with 58% and 88% when *t*_*BCB*_ = 0.95 μm and 0.55 μm, respectively. Remarkably, with the Meta-AR coating, the ER is improved by 30% as compared with only BCB coating, but on the contrary the thickness of BCB is reduced by 42%. The significant reduction of the thickness is beneficial in the low frequency regime (e.g. THz), where the thickness of AR coating is normally in the order of ~50 μm, which gives rise to great challenge in film deposition. The transmission at the second-order SPP resonance exhibits even higher ER with both BCB and Meta-AR coating due to the relatively low transmission for the bare MHA structure (as compared with *T*_*MHA*_ at the first-order SPP resonance). Accordingly, the maximum ERs are found to be 106% for BCB (*t*_*BCB*_ = 0.75 μm) coating and 99% for Meta-AR (*t*_*BCB*_ = 0.35 μm) coating (Fig. 3g). Note that the discrepancy between experiment and simulation for Meta-AR coating is probably due to the imperfections in the fabrication, specifically the misalignment between MDA and MHA (Discussions are provided in the Supporting Information).

### AR condition at SPP resonances

To understand the underlying mechanism of our Meta-AR coating, we developed a multiple-layer model based on a transfer matrix method^21^. Our Meta-AR coated MHA structure is composed of three layers: MDA, BCB and MHA on a GaAs substrate. Using a transfer matrix method, the overall reflection coefficient *r* of the three-layer structure can be obtained by multiplying the transfer matrix of each layer, *M* = *M*_1_ · *M*_2_ · *M*_3_, as given below and further details on the derivation are presented in the Supporting Information.


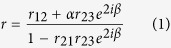


**Figure 4:**
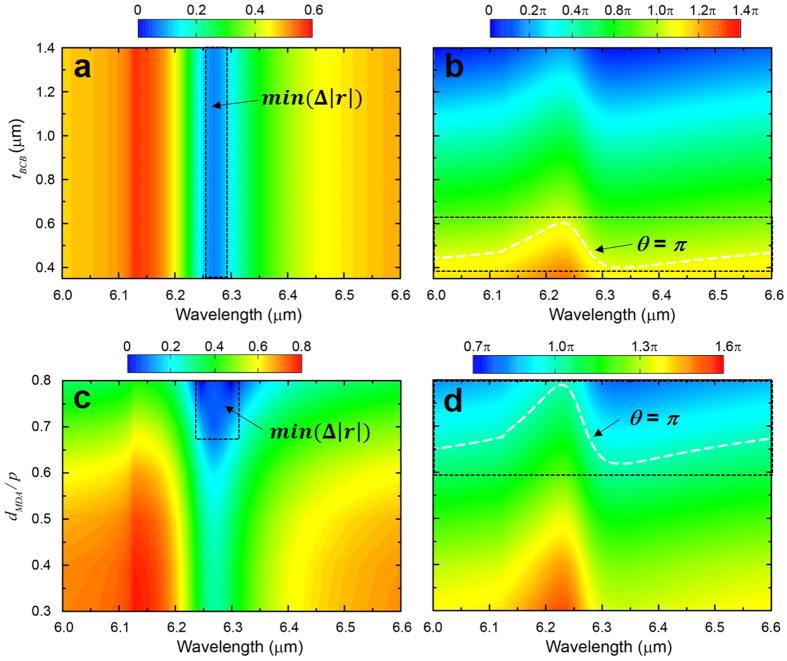
Colormaps of amplitude and phase AR-conditions around the first-order SPP resonance wavelength. Difference in amplitudes of reflection coefficients, 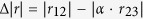 and the phase term, *θ* = *ϕ*(*r*_12_) − *ϕ*(*α* · *r*_23_) − 2*β* with (**a**,**b**) various BCB thicknesses and (**c**,**d**) MDA sizes, respectively.

The transmission and reflection coefficients involved in Eq. 1 can be obtained through numerical simulaitons. In particular, *r*_12_ and *r*_21_ are the reflection coefficients of MDA from front (air) and back (BCB) side, respectively. *r*_23_ is the reflection coefficient of the MHA. *β* = *n*_*BCB*_ · *k* · *t*_*BCB*_ is the propagating phase term in the BCB layer, where *n*_*BCB*_, *t*_*BCB*_ and *k* are the refractive index, the BCB thickness, and the wave vector in vacuum, respectively. *α* = *t*_21_*t*_12_ − *r*_21_*r*_12_, where *t*_12_ and *t*_21_ are the transmission coefficients through MDA along forward (air-MDA-BCB) and backward (BCB-MDA-air) directions, respectively. Although *α* is strictly equal to 1 at the interface between two homogenous media, *α* ≠ 1 for the MDA around the resonance wavelengths because of the structural asymmetry in forward and backward directions (air-MDA-BCB structure). In order to achieve perfect antireflection, *r* = 0 (Eq. 1) requires the following conditions for amplitude and phase:


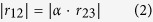



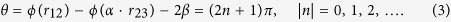


where the coefficients *r*_12_, *r*_21_, *t*_21_ and *t*_12_ are obtained from numerical simualtion of the air-MDA-BCB structure and *r*_23_ is obtained from the simulation of the BCB-MHA-GaAs structure. To find the appropriate geometric paramters of MDA that simultaneously satisfy amplitude (Eq. 2) and phase (Eq. 3) conditions, we carried out a series of simulations by varying the BCB thickness, *t*_*BCB*_ and the diameter for MDA, *d*_*MDA*_. Specifically, the complex reflection coefficients, *r*_12_ and *r*_23_, are obtained from simulations of two different configurations, air-MDA-BCB and BCB-MHA-GaAs, respectively. For the variation of BCB thickness, *d*_*MDA*_ is fixed at 0.7 · *p* (1.26 μm) and *t*_*BCB*_ is changed from 0.35 μm to 1.55 μm. For the variation of MDA size, *t*_*BCB*_ is fixed at 0.5 μm and *d*_*MDA*_ is changed from 0.3 · *p* (0.54 μm) to 0.8 · *p* (1.44 μm). The circular aperture size of MHA, *d*_*MHA*_ is kept at 0.5 · *p* (0.9 μm). Figure 4 shows the difference of amplutides, 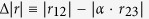
(Fig. 4a,c) and the phase term *θ* (Fig. 4b,d) for wavelengths around the first-order SPP resoannce. The regions enclosed by black rectangles represent the minimum value of Δ|*r*| and satisfaction of the phase condition (i.e. *θ* = *π*). Figure 4a suggests that the amplitudes of reflection coefficients, |*r*_12_| and |*r*_23_|, are independent of the BCB thickness for all *t*_*BCB*_ values in the range of 0.35 μm to 1.55 μm, so that Δ|*r*| can always reach the minimum value of Δ|*r*|, *min* (Δ|*r*|) = 0.081 at the first-order SPP resonance wavelength *λ*_1_ = 6.25 μm. The phase condition is satisfied when the BCB thickness is within a range of 0.35 μm < *t*_*BCB*_ < 0.6 μm around the white dash line as indicated in Fig. 4b. In combination, we find the amplitude and phase conditions are satisfied simultaneously when *t*_*BCB*_ ≈ 0.5 μm at the first-order SPP resonance wavelength *λ*_1_ = 6.25 μm. In contrast, the size of the MDA is strongly correlated to both the amplitude and phase as shown in Fig. 4c,d. In the region of 0.75 · *p* < *d*_*MDA*_ < 0.8 · *p*, both the amplitude Δ|*r*| ≈ 0 and the phase *θ* ≈ *π* conditions are satisfied simultaneously. Using the strategy of geometric parameter (*t*_*BCB*_, *d*_*MDA*_) sweep, we find the optimal AR coating structure with *t*_*BCB*_ = 0.5 μm and *d*_*MDA*_ = 0.78 · *p* (1.4 μm) that is able to completely eliminate the reflection at the first SPP resonance *λ*_1_ = 6.25 μm, and furthermore is capable of greatly reducing the reflection *R* at the second-order resonance *λ*_2_ = 4.38 μm. The optimized Meta-AR coating design (via our strategy) agrees well with the experimental results shown in Fig. 3; the fabricated sample with *t*_*BCB*_ = 0.55 μm and *d*_*MDA*_ = 0.7 · *p* shows the best transmission enhancement among all samples.

### Designer Metasurface (ε_eff_ and μ_eff_) using thin-film AR coating mechanism

Due to the small size-to-wavelength ratio, *d*_*MDA*_/*λ* ~ 0.1667, the role of MDA in the Meta-AR coating can be understood by using the effective medium theory. The MDA exhibits localized surface plasmon resonances at wavelengths determined by its geometric size and the refractive indices of surrounding materials^14^. The plasmon resonances induce abrupt amplitude and phase changes to the light reflected by or transmitted through the MDA layer. Such phase and amplitude discontinuities have been used to reshape the beam profile to achieve negative refraction and beam steering^22^,^23^. These single layers of plasmonic resonator arrays are typically regarded as two-dimensional metasurfaces instead of bulk metamaterial since they only consist of single “meta-atom” layer. The electromagnetic properties of the metasurface can be described by the effective surface electric susceptibility *χ*_*se*_ and the effective surface magnetic susceptibility *χ*_*sm*_, which are calculated from the transmission and reflection coefficients^24^,^25^. Due to the skin effect, the induced electric current in the plasmonic resonators only flows within extreme thin region under the surface of the structure (in the order of the skin depth 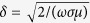, where *σ* is the conductivity of metal). Therefore, *χ*_*se*_ and *χ*_*sm*_ are typically independent of the physical thickness of the resonators. However, at infrared regime, the conductivity of metal decreases dramatically, so that the skin effect is less pronounced and the electric current distributes throughout an entire volume of the resonator. As a results, we observe that the surface susceptibilities *χ*_*se*_ and *χ*_*sm*_ increase as the thickness of the MDA increases (see Supporting Information). To address the thickness dependence, in this work we model the MDA metasurface as a thin film (metafilm) with the same thickness as the MDA (*t*_*MDA*_). The effective permittivity *ε*_*eff*_ and effective permeability *μ*_*eff*_ of the metafilm can be calculated from simulated transmission and reflection coefficients of an air-MDA-BCB configuration by using a well-known retrieval method^26^ or alternatively by 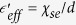 and 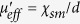
24,25. With *ε*_*eff*_ and *μ*_*eff*_, the metafilm model considers the MDA layer as an effective thin film, which provides a more intuitive description compared to effective surface susceptibilities *χ*_*se*_ and *χ*_*sm*_. It can be shown that the phase of EM waves transmitted through and reflected by the metafilm is exactly equal to the abrupt phase changes of the corresponding transmission and refleciton coefficients of the actual MDA metasurface (see Supporting Information). Using the metafilm model, the meta-AR coating can be understood intuitively as the classical thin-film AR coating that consists of two thin flims, i.e. MDA metafilm and BCB layer.

**Figure 5:**
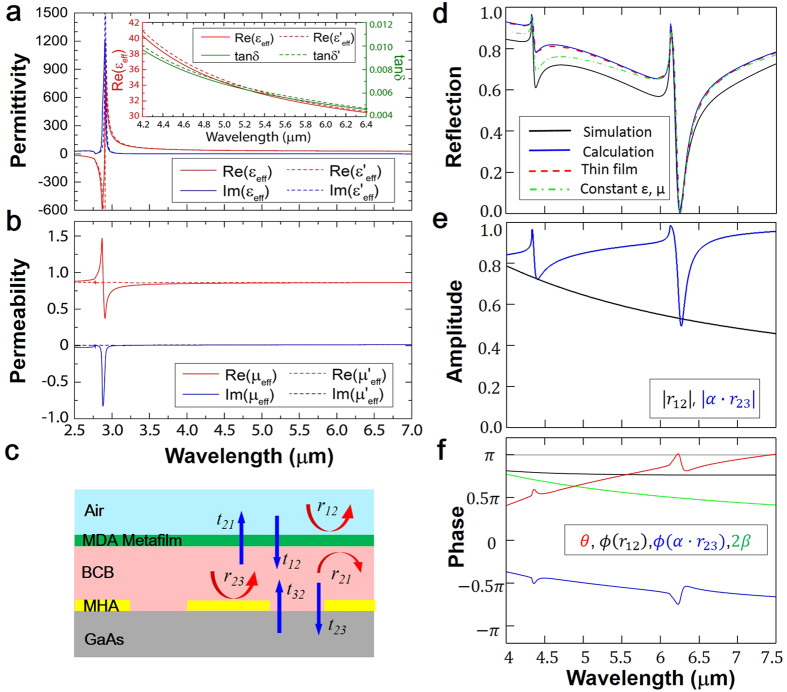
The real (red) and imaginary (blue) parts of (**a**) effective permittivity *ε*_*eff*_ and (**b**) permeability *μ*_*eff*_ of the MDA metasurface. Inset shows the real part of effective permittivity, *Re*(*ε*_*eff*_) (red) and the loss tangent, tan *δ* (green). (**c**) Diagram of the reflection and transmission coefficients of MDA and MHA. (**d**) Reflections obtained by full-wave simulation of actual MDA-BCB-MHA (black line), analytical calculation (blue line) using Eq. 1 and full-wave simulation of metafilm-BCB-MHA structure with wavelength-dependent (red dash line) and wavelength-independent (green dash-dot line) effective parameters. (**e**) The amplitude and phase (**f**) terms in the AR conditions (Eqs 2 and 3).

Both the effective surface susceptibilities, *χ*_*se*_ and *χ*_*sm*_ in the metasurface model^24^,^25^ and *ε*_*eff*_ and *μ*_*eff*_ in the retrieval method^26^ are calculated using the transmission and reflection coefficients of an air-MM-air configuratoin. In reality, MM structures are usually made on a dielectric substrate, so the transmission and reflection coefficients are obtained at the interfaces of an air-MM-dielectric configuration. To the best of our knowledge, many MM related works use the transmission and reflection coefficients of air-MM-dielectric configuration directly to extract *ε*_*eff*_ and *μ*_*eff*_, which cause inaccurate values. In our structure, the metasurface is modeled as a metafilm with thickness *t*_*MDA*_ = 50 nm. The extremely thin thickness magnifies the error greatly because the effective refractive index of the metafilm, *n*_*eff*_, is inversely proportional to *t*_*MDA*_. To obtain correct effective parameters of the metafilm, we develop a method to obtain the transmission and reflection coefficients of the air-MM-air configuraiton from that of the air-MM-dielectric configuation. The resutlsing transmission and reflection coefficients produce accurate *χ*_*se*_, *χ*_*sm*_, *ε*_*eff*_ and *μ*_*eff*_. Details of this improved retrieval method are elucidated in the Supporting Information. Figure 5a,b show *ε*_*eff*_ and *μ*_*eff*_ of MDA structure calculated from simulation data of an air-MDA-BCB configuration with *t*_*BCB*_ = 0.5 μm and *d*_*MDA*_ = 0.78 · *p*, where solid lines show *ε*_*eff*_ and *μ*_*eff*_ calculated by the retrieval method and dash lines show 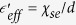 and 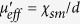. It can be seen that two methods give nearly identical values except minor deviation at the resonance wavelength *λ*_*r*_ = 2.92 μm, where the thin-film retrieval shows typical antiresonance in *μ*_*eff*_ due to the periodicity effects^27^. The strong Lorentzian line shaped resonance in *ε*_*eff*_ indicates an electric response, where the electric field of the incident light induces resonant electric current in the disk. *μ*_*eff*_ is nearly constant (*μ*_*eff*_ ≈ 0.86) within the entire wavelength range from 2.5 μm to 7.0 μm. The resonance wavelength (*λ*_*r*_ = 2.92 μm) of MDA is much shorter than the operating wavelengths of our Meta-AR coating, i.e. the first-order (*λ*_1_ = 6.25 μm) and second-order (*λ*_2_ = 4.38 μm) SPP resonances of MHA. Therefore, the imaginary parts (blue lines) of *ε*_*eff*_ and *μ*_*eff*_ at these wavelengths are nearly zero, and hence the loss in MDA is neglegible. Specifically, as shown in the inset of Fig. 5a, the real part of *ε*_*eff*_ gradually decreases from 40.25 to 30.52 in the wavelength range from 4.2 μm to 6.4 μm. The loss tangent, defined as the ratio between the imaginary and the real parts of the permittivity (tan *δ* = *ε*^′′^/*ε*^′^), also decreases from 0.0097 to 0.0046. The effective permittivity reads values of 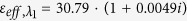 and 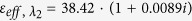 at the first-order and second-order SPP resonance wavelengths (*λ*_1_, *λ*_2_), respectively. Note that the loss tangents, 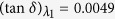 and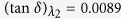, have the same order of magnitude of commonly used low-loss dielectric coating materials such as polyimide (tan *δ*~0.005) and BCB (tan *δ*~0.001).

With the calculated *ε*_*eff*_ and *μ*_*eff*_, we can replace the MDA layer as a thin film of thickness *t* = *t*_*MDA*_. As illustrated in Fig. 5c, the AR coating on the MHA comprises two layers of homogenous films: a MDA metasurface with *ε*_*eff*_ and *μ*_*eff*_ and a BCB layer. The numerically calculated (blue line: using Eq. 1) and simulated reflections (black line: using actual MDA structure; red dash and green dash-dot lines: using homogeneous film with wavelength-dependent (*ε* = *ε*_*eff*_(*λ*), *μ* = *μ*_*eff*_(*λ*) as shown in Fig. 5a,b) and wavelength-independent (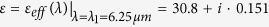, 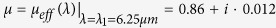) effective parameters, respectively) are shown in Fig. 5d. At the first-order SPP resonance (*λ*_1_ = 6.25 μm), all four curves are matched very well and perfect antireflection is achieved with nearly zero reflection (*R* ≈ 0). Such perfect match is attributed to the fact of *λ*_1_ ≫ *λ*_*r*_, so that the MDA operates at off-resonance wavelengths. In this region, the MDA layer only transmits/reflects light without any resonant coupling with MHA layer. Therefore, the calculation (blue) based on the transfer matrix method (Eq. 1) can perfectly reproduce the full-wave simulation of actual MDA-Spacer-MHA structure (black). The simulations using metafilms with dispersive (red dash) and constant (green dash-dot) *ε*_*eff*_ and *μ*_*eff*_ show that these effective parameters correctly discribe the EM property of MDA layer in the MDA-spacer-MHA system. However, at the second-order SPP resonance, the simulated reflection using effective medium model does not match with using actual MDA structure. The discrepancy attributes to the resonance coupling between the MHA and the MDA (Supporting Information) because the second-order SPP resonance (*λ*_2_ = 4.38 μm) is much closer to the resonance wavelength (*λ*_*r*_ = 2.92 μm) of the MDA (Note: the effective permittivity and permeability of MDA are obtained from simulation of the air-MDA-BCB configuration, where the coupling between the MDA and the MHA resonances is absent). The phase and amplitude conditions given in Eqs 2 and 3 reveal the underlying mechanism of AR as the destructive interference of light reflected at the MDA-BCB and BCB-MHA interfaces. In Fig. 5e, we can clearly see that the amplitudes of *r*_12_ and *α* · *r*_23_ are equal at the first-order SPP resonance wavelength *λ*_1_ = 6.25 μm (i.e. the amplitude condition is satisfied). Simultaneously, as shown in Fig. 5f, the phase term, *θ* = *ϕ*(*r*_12_) − *ϕ*(*α* · *r*_23_) − 2*β* crosses *π* (gray line), as predicted in the phase condition. At the second-order SPP resonance *λ*_2_ = 4.38 μm, the reflection is reduced but does not reach zero because only the amplitude condition is satisfied.

## Discussion

In summary, we have experimentally, numerically and analytically investigated the enhanced transmission due to a metasurface antireflection (AR) coating on a dispersive surface plasmon (SP) structure in the mid-infrared regime. Our metasurface AR coating (based on MDA) works at off-resonance wavelengths and can be modeled as a metafilm with high effective permittivity (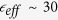). The extremely low loss tangent, 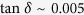, is comparable to low-loss films used in AR coating such as polyimide (tan *δ*~0.005) and BCB (tan *δ* ~ 0.001). In addition, the effective permittivity *ε*_eff_ is easily tunable by changing the geometric size of the MDA, which provides unprecedented flexibility to fit the different wavelengths for a variety of applications. With the metasurface coating, the measured transmission through the dispersive SP structure is greatly increased at both the first-order (58% for *t*_*BCB*_ = 0.35 μm; 88% for *t*_*BCB*_ = 0.55 μm) and second-order (99% for *t*_*BCB*_ = 0.35 μm; 80% for *t*_*BCB*_ = 0.55 μm) SP resonances. The electric field and the intensity of surface wave are also enhanced by ~33% and ~77%, respectively, for the first-order SP resonance. The enhanced electric field of surface wave will benefit to applications where the local field engineering (strong local field) is demanded, e.g. improving the performance of optoelectronic devices. Moreover, the metafilm model, transfer matrix analysis and improved retrieval method developed in our work are generally applicable to multi-layered metasurface system including antireflection coating and plasmonic perfect absorbers^21^,^28^,^29^.

## Additional Information

**How to cite this article**: Jeon, J. *et al.* A Low-loss Metasurface Antireflection Coating on Dispersive Surface Plasmon Structure. *Sci. Rep.* **6**, 36190; doi: 10.1038/srep36190 (2016).

**Publisher’s note:** Springer Nature remains neutral with regard to jurisdictional claims in published maps and institutional affiliations.

## Supplementary Material

Supplementary Information
