# Supplementary material for: A Low-loss Metasurface Antireflection Coating on Dispersive Surface Plasmon Structure
Source: Sci Rep. 2016 Nov 2;6:36190. doi: 10.1038/srep36190 (PMC5090358; doi:10.1038/srep36190)
Supplement: Supplementary Information [file srep36190-s1.pdf]

Supporting Information:

A Low-loss Metasurface Antireflection Coating on Dispersive Surface Plasmon Structure

Jiyeon Jeon, Khagendra Bhattarai, Deok-Kee Kim, Jun Oh Kim, Augustine Urbas, Sang Jun Lee, Zahyun Ku and Jiangfeng Zhou

Fabrication procedure of Meta-AR coated MHA

The processing scheme of the Meta-AR coated MHA were as follows. (I) Hexamethyldisilazane (HMDS) as an adhesion layer was spread atop a semi-insulating double polished GaAs (1 × 1 cm^2) substrate. (II) AZ 5206 photoresist was spun onto a substrate at 5K rpm for 50 s and hotplate-baked at 100°C for 60 s. (III) The Kark Suss MA6 mask aligner was carried out to produce a periodic circular post pattern in the photoresist layer with image reversal process (specifically, first exposure was performed with photomask for 23 s; the reversal bake on 112°C hotplate was used for 90 s; the flood exposure was done for 100 s; the reversal-baked and flood-exposed sample was developed with AZ340 for 90 s and rinsed with deionized water). (IV) After the PR pattern was defined, consecutive e-beam evaporations were used to deposit a 5-nm thick layer of titanium (Ti) as an adhesion on the developed sample and a 50-nm thick layer of gold (Au) at a pressure of the order of 10^{-7} Torr during deposition and ~0.02 nm/s, followed by a lift-off processing with a acetone to remove the PR layer. This resulted in MHA (p = 1.8 μm, $d_{MHA} = 0.5 \cdot p$) on GaAs substrate as shown in Figure S1. (V) BCB was spin-coated on the fabricated MHA sample with reference of experimental BCB thickness as a function of spin-coating speed and dilution ratio between

BCB and rinse solvent (Cyclotene 3022-35 and T1100, The Dow Chemical Company), then was cured in a vacuum oven at 250°C for 1 hr, leading to a flat-top BCB coated MHA sample (flat and smooth top-surface is obviously shown between MHA and MDA as shown in Figure S1). (VI) Next, a periodic circular hole pattern in the positive-tone PR (AZ 5214) was defined on BCB layer (p = 1.8 μm, d_{MDA} = 0.7·p), followed by e-beam deposition of 50 nm of Au. Note that the aperture size was adjusted by the exposure and development times. (VII) An acetone was again used to remove the PR layer, leading to a final structure, Meta-AR coated MHA on GaAs (MHA coated with an array of circular gold disks atop the BCB layer) as shown in Figure S1.

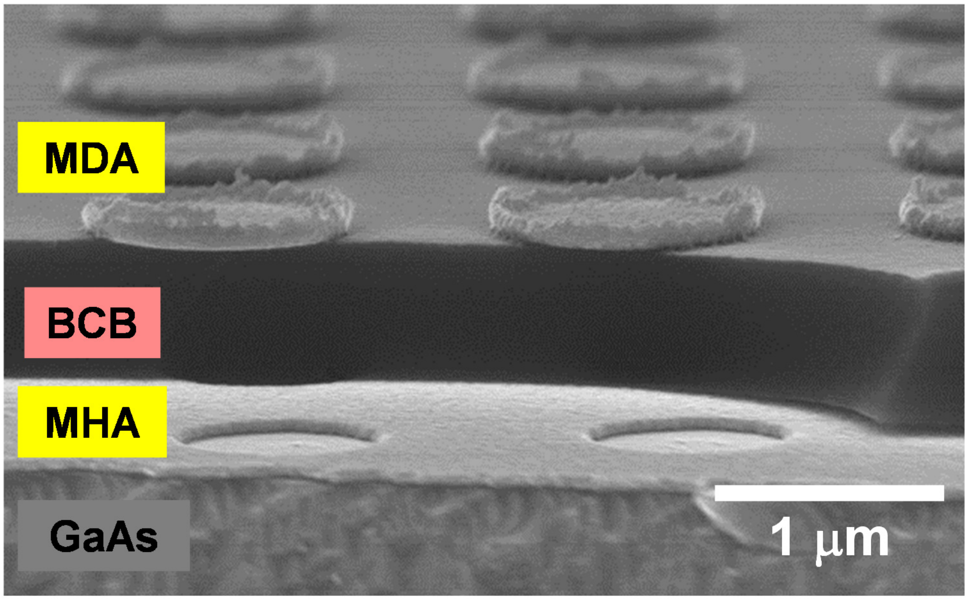

Figure S1. SEM image of completed Meta-AR coated MHA sample

Transfer Matrix Analysis

In our Meta-AR (MDA atop BCB layer) coated MHA structure, the incident light is reflected at the two interfaces; MDA/BCB and BCB/MHA. Zero reflection owing to Meta-AR coating layer

can be interpreted by using a multiple-layer model in which each layer is considered as homogeneous meta-layer with effective electric permittivity ε_{eff} and magnetic permeability μ_{eff}. A transfer matrix method is adopted to obtain the overall transmission and reflection coefficients using a transfer matrix of each layer. The transfer matrix of the air-MDA-BCB structure can be described by

$$M_1 = \begin{pmatrix} M_{11} & M_{12} \\ M_{21} & M_{22} \end{pmatrix} = \begin{pmatrix} t_{21} - r_{12}r_{21}/t_{12} & r_{12}/t_{12} \\ -r_{21}/t_{12} & 1/t_{12} \end{pmatrix} \quad \text{(S1)}$$

where r_{12}, r_{21}, t_{12} and t_{21} are the transmission and reflection coefficients obtained from the numerical simulation. The transmission coefficient t_{ij} is defined as the S-parameter S_{ji} and the reflection coefficient r_{ij} is defined as the S-parameter S_{ii}, where the subscript i (j) represents the waveguid port at i-material (j-material). Specifically, t_{12} and t_{12} are the transmission coefficients along forward (air-MDA-BCB) and backward (BCB-MDA-air) directions, respectively. r_{12} and r_{21} are the reflection coefficients at front (air) and back (BCB) sides of MDA. The transfer matrix for the BCB layer is given by

$$M_2 = \begin{pmatrix} e^{i\beta} & 0 \\ 0 & e^{-i\beta} \end{pmatrix} \quad \text{(S2)}$$

where β is the propagating phase term in the BCB layer ($\beta = n_{BCB} \cdot k \cdot d_{BCB}$: n_{BCB} is the refractive index of the BCB, k is the wave vector in vaccum and d_{BCB} is the thickness of the BCB layer). Also, the transfer matrix for the BCB-MHA-GaAs configuration can be written as given below,

$$M_3 = \begin{pmatrix} M_{11} & M_{12} \\ M_{21} & M_{22} \end{pmatrix} = \begin{pmatrix} t_{32} - r_{23}r_{32}/t_{23} & r_{23}/t_{23} \\ -r_{23}/t_{23} & 1/t_{23} \end{pmatrix} \quad \text{(S3)}$$

where the subscript 2(3) represents the waveguid port at BCB (GaAs). As mentioned above,

the overall reflection coefficient of Meta-AR coated MHA on GaAs substrate can be calculated by multiplying the transfer matrix of each layer,

$$M = M_1 M_2 M_3$$

$$= \begin{pmatrix} t_{21} - \frac{r_{12}r_{21}}{t_{12}} & \frac{r_{12}}{t_{12}} \\ \frac{-r_{21}}{t_{12}} & \frac{1}{t_{12}} \end{pmatrix} \cdot \begin{pmatrix} e^{i\beta} & 0 \\ 0 & e^{-i\beta} \end{pmatrix} \cdot \begin{pmatrix} t_{32} - \frac{r_{23}r_{32}}{t_{23}} & \frac{r_{23}}{t_{23}} \\ \frac{-r_{23}}{t_{23}} & \frac{1}{t_{23}} \end{pmatrix}$$

$$= \begin{pmatrix} \left(t_{21} - \frac{r_{12}r_{21}}{t_{12}}\right) \cdot \left(e^{i\beta}\right) \cdot \left(\frac{r_{23}}{t_{23}}\right) + \left(\frac{r_{12}}{t_{12}}\right) \cdot \left(e^{-i\beta}\right) \cdot \left(\frac{1}{t_{23}}\right) \\ \left(\frac{-r_{21}}{t_{12}}\right) \cdot \left(e^{i\beta}\right) \cdot \left(\frac{r_{23}}{t_{23}}\right) + \left(\frac{1}{t_{12}}\right) \cdot \left(e^{-i\beta}\right) \cdot \left(\frac{1}{t_{23}}\right) \end{pmatrix}$$

$$= \begin{pmatrix} \frac{(t_{21}t_{12} - r_{12}r_{21}) \cdot e^{i\beta} \cdot r_{23} + r_{12} \cdot e^{-i\beta}}{t_{21}t_{23}} \\ \frac{-r_{21}r_{23} \cdot e^{i\beta} + e^{-i\beta}}{t_{21}t_{23}} \end{pmatrix}$$

$$\therefore r = \frac{\frac{(t_{21}t_{12} - r_{12}r_{21}) \cdot e^{i\beta} \cdot r_{23} + r_{12} \cdot e^{-i\beta}}{t_{21}t_{23}}}{\frac{-r_{21}r_{23} \cdot e^{i\beta} + e^{-i\beta}}{t_{21}t_{23}}}$$

$$= \frac{(t_{21}t_{12} - r_{12}r_{21}) \cdot e^{i\beta} \cdot r_{23} + r_{12} \cdot e^{-i\beta}}{-r_{21}r_{23} \cdot e^{i\beta} + e^{-i\beta}}$$

$$= \frac{r_{12} + (t_{21}t_{12} - r_{12}r_{21}) \cdot r_{23} \cdot e^{2i\beta}}{1 - r_{21}r_{23} \cdot e^{2i\beta}}$$

$$= \frac{r_{12} + \alpha \cdot r_{23} \cdot e^{2i\beta}}{1 - r_{21}r_{23} \cdot e^{2i\beta}} \tag{S4}$$

where $\alpha = t_{21}t_{12} - r_{12}r_{21}$.

Coupling effects between MDA and MHA

Reflection obtained by a finite integration technique based simulation of the entire structure

(Meta-AR coated MHA on GaAs structure) and the calculation using Equation. S4 match very well at the first-order SPP resonance as shown in Figure 5d. However, they are not identical across the whole wavelength region for interest. The discrepancy is clrealy seen around the second-order SPP resonance, giving rise to the resonance of the MDA. Since the MDA resonance wavelength ($\lambda_r = 2.92$ μm as shown Figure 5a) is close to the second-order SPP resonance of MHA ($\lambda_2 = 4.38$ μm), two resonances of MHA and MDA couple each other. Thereby, the calculated total reflection deviats from the simulation of whole structure. The multiple-layer model does not cosider the coupling between different layers consisting of the Meta-AR coated MHA structure. In our struture, we have two metallic resonators; MHA and MDA separated by BCB. When two metallic layers are far enough so as not to couple each other, the calculated reflection using Equation S4 match completely with the simulation of the entire structure. When the BCB thickness is thin enough to couple the resonances of MHA and MDA, the simulated reflection will be changed. In order to support our expectation, we have calculated the total reflection using Equation S4 for three different thicknesses of BCB (t_{BCB} = 0.25 μm, 0.5 μm and 0.95 μm) and then compared with the simulation of the corresponding entire structure (Figure S2) with the parameters used in the simulation as follows: Periodicity (p) = 1.8 μm, Diameter of MHA (d_{MHA}) = $p/2$, diameter of MDA (d_{MDA}) = 0.78·p, permittiviy of GaAs (ε_{GaAs}) = 11.56 , permittivity of BCB (ε_{BCB}) = 2.37, and thickness of gold (t_{Au}) =0.05 μm. It can be noticed from Figure S2 that the calculated (red dash-dot line) and simulated (red solid line) reflections very closely match for the BCB thickness (t_{BCB}) of 0.95 μm. As the BCB thickness decreases, the mismatch between simulation and calcualtion is more likely to suffer. As the two metal layers come closer, their field couple each other changing the effective permittivity thereby changing the reflective behavior.

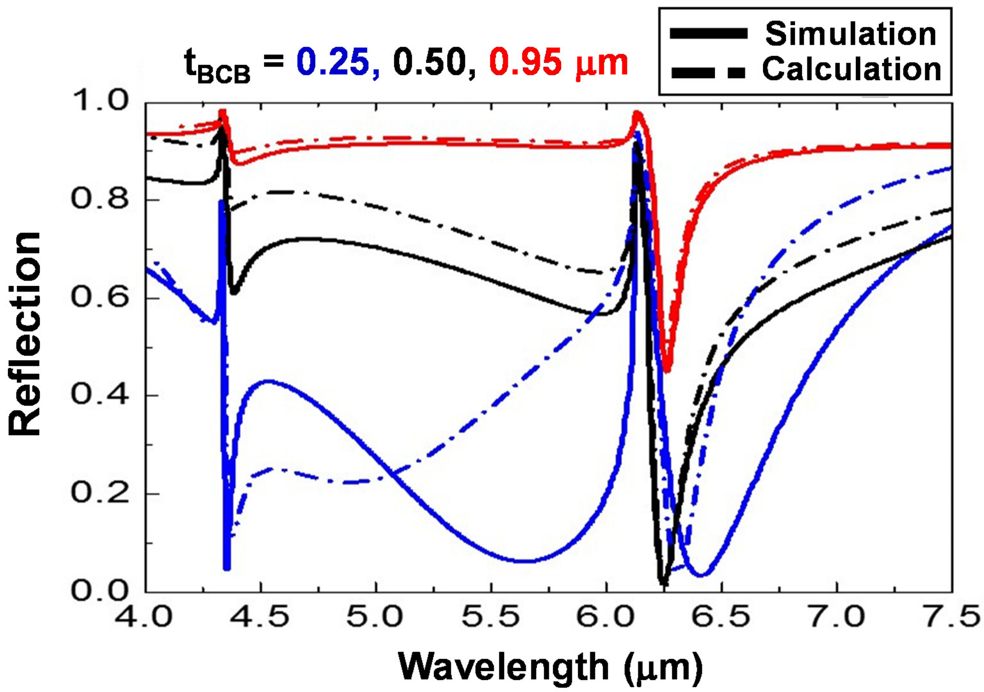

Figure S2. Simulated reflection of the entire structure and numerical calculated reflection using the multiple-layer model (Equation S4) for p = 1.8 μm and d_{MDA} = 0.78·p. Solid lines refer to the simulation and dash-dot lines refer to the calculation. Blue, black and red lines represent the BCB thickness of 0.25 μm, 0.5 μm and 0.95 μm, respectively.

Misalignment between MDA and MHA due to fabrication imperfection

For ideal fabrication conditions, the transmission could be improved owing to the Meta-AR coating (p=1.8 μm, d_{MDA} = 1.26 μm, t_{BCB} = 0.35 μm) as high as about 82% and 174% for the first-order and second-order SPP resonance, as compared with the transmission of MHA. In reality, the quantitative disagreement between the experimentally measured and simulated enhancement ratio was obtained as shown in Figure 3d,e (Note that the trends of simulation and experiment results show good agreement), which is probably due to imperfections in the fabrication. To better understand our fabrication imperfections (SEM image of Figure S3b) qualitatively, we performed 3D full field EM simulations of Meta-AR coated MHA using a finite

integration technique. The Meta-AR coated MHA structure is composed of MHA coated with MDA atop BCB layer, but the circular Au disks (in MDA) are misaligned with the circular holes (in MHA). As illustrated in Figure S3a, the misalignment is defined as the distance (in x- and y-direction) from the center of MHA aperture to the center of MDA disk, (x, y). For perfect alignment between MDA and MHA, i.e. for ideal fabrication (x, y) must be (0, 0). Initially, the frequency domain solver using the full Floquet modal expansion, periodic boundaries in the x- and y-directions and open boundary condition in z-direction (perfectly matched layers) was used to calculate the S-parameters of the unit cell with various combination of (x, y): 0.3 μm $(0.17 \cdot p) \leq \mathrm{x}, \mathrm{y} \leq$ 0.9 μm $(0.5 \cdot p)$. Note that x or y of 0.9 μm can be considered as fully misaligned MDA. The transmission enhancement ratio map is presented in color as a function of x and y as displayed in Figure S3c,d. The misalignment (x, y) is varied continuously and the color changes from blue to red for increased enhancement ratio (44% to 77% at $\lambda_1 =$ 6.25 μm and -23% to 159% at $\lambda_1 =$ 4.38 μm for the first- and second-order SPP resonances, respectively). The black dash lines indicate ±15% error range of measured enhancement ratio (See Figure 3d,e). We found that (x, y) = (0.5, 0.5) resulted in the enhancement ratio close to experimental values for both first-order and second-order SPP resonances, i.e. the MDA could be fabricated with as much shift as about 28% in x- and y-directions, compared with the perfect alignment. The lower measured enhancement ratio is due to the misalignment between MDA and MHA resulting from fabrication imperfections.

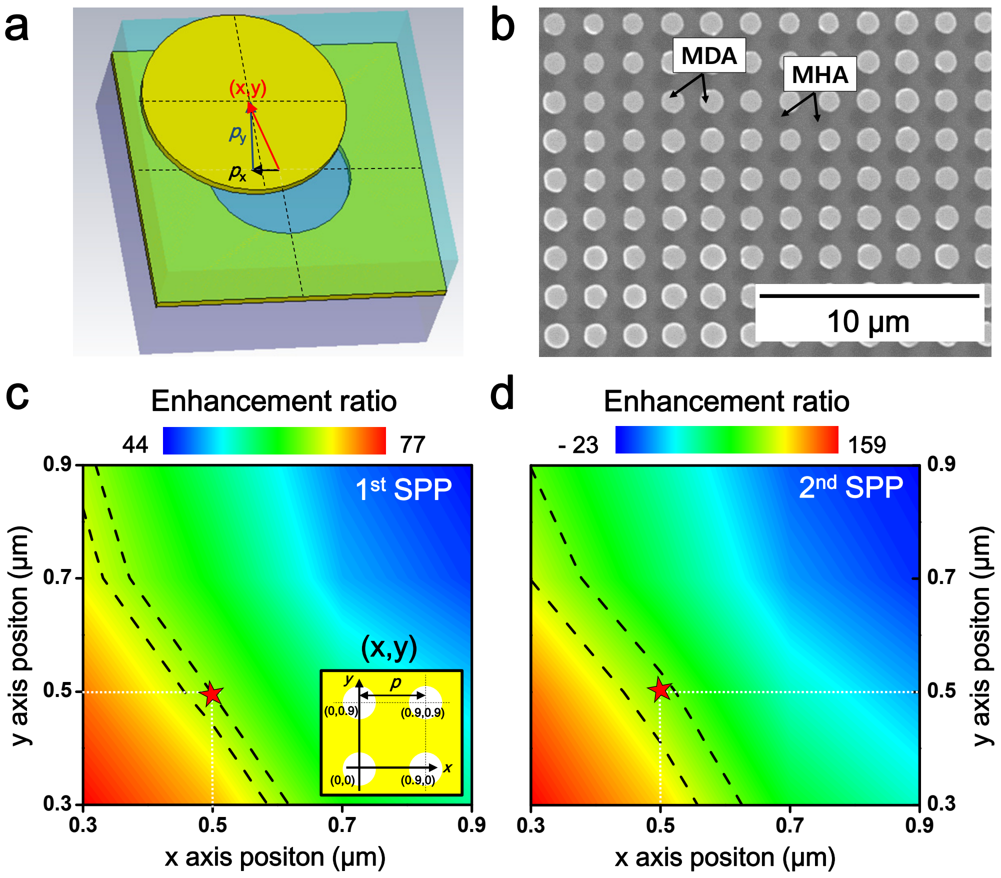

Figure S3. (a) Schematic view of unit cell of Meta-AR coated MHA structure. (b) SEM image showing the misaligned MDA with MHA. Colormaps of simulated enhancement ratio as a function of misaligned MDA position in *x*- and *y*-direction for (c) the first-order (λ_1 = 6.25 μm) and (d) the second-order (λ_2 = 4.38 μm) SPP resonances. Note that we performed the FIT based simulation (CST Microwave Studio) with p = 1.8 μm, d_{MHA} = 0.9 μm, t_{Au} = 0.05 μm, and d_{MDA} = 1.26 μm.

Metafilm model

The disk-type plasmonic resonator can be considered as a metasurface since the induced electric current only resides at the surface of the structure due to the skin effect. The electromagnetic properties of the metasurface can be described by the effective surface electric susceptibility χ_{se} and the effective surface magnetic susceptibility χ_{sm}, which can be

calculated from the transmission and reflection coefficients[1,2]. For normal incidence, χ_{se} and χ_{sm} can be calculated by the following equations:

$$\chi_{se} = \frac{2i}{k}\frac{1-r-t}{1+r+t} \quad \text{(S5)}$$

$$\chi_{sm} = \frac{2i}{k}\frac{1+r-t}{1-r+t} \quad \text{(S6)}$$

where k is the wavevector in vacuum, and t and r are complex transmission and reflection coefficients, respectively. Typically, χ_{se} and χ_{sm} do not change with physical thickness of the metallic resonant structure. However, in the infrared regime, the conductivity of metal decreases diametrically, so that the electric current flows through the entire volume of the metallic structure. Thus, χ_{se} and χ_{sm} show clear dependence on the thickness of the disk t_{MDA}, as shown in Figure S4a,b. In this work, since the electromagnetic properties of the disk-type resonator depend on the thickness, we model it as a thin film (metafilm) with effective permittivity ϵ_{eff}, and effective permeability μ_{eff}. ε_{eff} and μ_{eff} of the metafilm can be calculated by using the well-known retrieval method[3] or by the relations of $\varepsilon'_{eff} = \chi_{se}/d$ and $\mu'_{eff} = \chi_{sm}/d$. As shown in Figure. S4c,d, ε_{eff} and μ_{eff} calculated by two methods match very well. The retrieval method using the following equations to calculate ε_{eff} and μ_{eff}:

$$z_{eff} = \pm\sqrt{\frac{(1+r)^2-t^2e^{2ikd}}{(1-r)^2-t^2e^{2ikd}}} \quad \text{(S7)}$$

$$Re(n_{eff}) = \pm Re\left(\frac{\cos^{-1}\left(\frac{1}{2te^{ikd}}\left[1-\left(r^2-t^2e^{2ikd}\right)\right]\right)}{kd}\right) + \frac{2m\pi}{kd} \quad \text{(S8)}$$

$$Im(n_{eff}) = \pm Im\left(\frac{\cos^{-1}\left(\frac{1}{2te^{ikd}}\left[1-\left(r^2-t^2e^{2ikd}\right)\right]\right)}{kd}\right) \quad \text{(S9)}$$

$$\varepsilon_{eff} = n_{eff}/z_{eff} \quad \text{(S10)}$$

$$\mu_{eff} = n_{eff} z_{eff} \tag{S11}$$

where z_{eff} and n_{eff} are the effective impedance and effective refractive index, respectively, and d is the thickness of the metamaterial. The sign of z_{eff} and n_{eff} are determined respectively by the fact that $Re(z_{eff}) > 0$ and $Im(n_{eff}) > 0$ for any passive medium. The integer m in Equation S8 is usually determined by the continuity of $n_{eff}(\lambda)$ as a function of the wavelength.

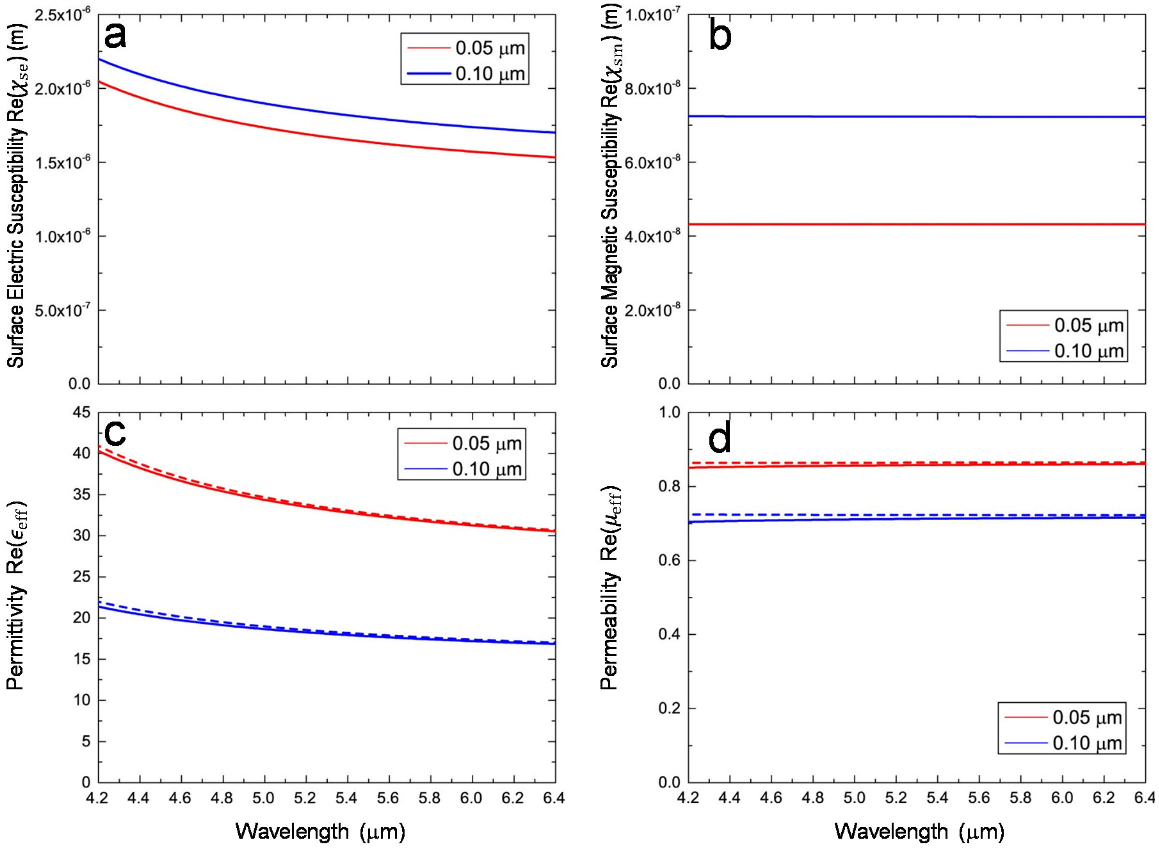

Figure S4. (a),(b) Real part of χ_{se} and χ_{sm} of the MDA metasurface for the thickness $t_{MDA} = 0.05\ \mu m$ (Red) and $t_{MDA} = 0.10\ \mu m$ (blue). (c),(d) Real part of ϵ_{eff} and μ_{eff} of the MDA metafilm, where the solid curves are calculated by the retrieval method and the dashed curves are calculated by χ_{se}/t_{MDA} and χ_{sm}/t_{MDA}.

Both the metasurface model Equations S5 & S6 and the effective retrieval method Equations S7-11 assume that the metamaterial is bounded by air at both sides forming an air-MM-air configuration. However, in reality MM structures are usually made on a dielectric substrate. The transmission and reflection coefficients are obtained at the interfaces of an air-MM-dielectric configuration as shown in Figure S5b. To the best of our knowledge, most works related to MM use the transmission and reflection coefficients of air-MM-dielectric configuration directly to calculate ε_{eff} and μ_{eff}, which cause inaccurate values. In our structure, the metasurface is modeled as a metafilm with thickness $d = 50\,nm$. The extremely thin thikness d magnifies error signaficantly because d exists in the denominator in Equations S8 & S9. To obtain the correct ε_{eff} and μ_{eff}, we develop a method to obtain the transmission and reflection coefficients of the air-MM-air configuraiton (Figure S5c), $t'_{12}, t'_{21}, r'_{12}$ and r'_{21}, from t_{12}, t_{21}, r_{12} and r_{21} of the air-MM-dielectric configuation (Figure S5b). The results of χ_{se}, χ_{sm}, ε_{eff} and μ_{eff} shown in Figure S4 & S5 are calculated from $t'_{12}, t'_{21}, r'_{12}$ and r'_{21} of the air-MM-air configuration by using this improved metasurface model and effective thin-film retrieval method. The transfer matrix at the interface of two dielectric media with impedance z_1 and z_2 is given by

$$M_{12} = \frac{1}{2z_2}\begin{pmatrix} z_2 + z_1 & z_2 - z_1 \\ z_2 - z_1 & z_2 + z_1 \end{pmatrix}$$

Therefore, the transfer matrix of the air-MM-dielectric configuration can be written as

$$M_{AMD} = M_1 M_2 M_3 \tag{S12}$$

$$M_1 = \frac{1}{2z_M}\begin{pmatrix} z_M + z_0 & z_M - z_0 \\ z_M - z_0 & z_M + z_0 \end{pmatrix} \tag{S13}$$

$$M_2 = \begin{pmatrix} e^{in_M kd} & 0 \\ 0 & e^{-in_M kd} \end{pmatrix} \tag{S14}$$

$$M_3 = \frac{1}{2z_D}\begin{pmatrix} z_D + z_M & z_D - z_M \\ z_D - z_M & z_D + z_M \end{pmatrix} \tag{S15}$$

where z_M and n_M are the effective impedance and effective refractive index of the MM, respectively, k is the wave number in vaccum, z_0 and z_D are the impedance of air ($z_0 = 1$) and dielectric ($z_D = 1/n_D$) layer, respectively. If we insert an infnite-thin ($d = 0$) air layer between the MM and the dielectric layer, the transfer matrix of the resulting air-MM-air-dielectric configuration is given by

$$M_{AMAD} = M_1 M_2 (M_{M0} M_{0D})$$

where M_{M0} and M_{0D} are the transfer matrices of the MM-air and air-dielectric interfaces, respectively

$$M_{M0} = \frac{1}{2z_0}\begin{pmatrix} z_M + z_0 & z_0 - z_M \\ z_0 - z_M & z_0 + z_M \end{pmatrix}$$

$$M_{0D} = \frac{1}{2z_D}\begin{pmatrix} z_D + z_0 & z_D - z_0 \\ z_D - z_0 & z_D + z_0 \end{pmatrix}$$

It is easy to prove that

$$M_{M0} M_{0D} = \frac{1}{2z_D}\begin{pmatrix} z_M + z_D & z_D - z_M \\ z_D - z_M & z_D + z_M \end{pmatrix} = M_3$$

Thus we obtain

$$M_{AMAD} = M_1 M_2 (M_{M0} M_{0D}) = M_1 M_2 M_3 = M_{AMD}$$

Note that this result proves inserting an infinite-thin air layer does not change the transfer matrix of the overal all structure. So that

$$M_{AMA} = M_1 M_2 M_{M0} = (M_1 M_2 M_{M0} M_{0D})(M_{0D})^{-1} = M_{AMD}(M_{0D})^{-1} \quad \text{(S16)}$$

The matrix M_{AMA} is the transfer matrix for the air-MM-air configuration (Figure S5c). In Equation S16, M_{AMD} can be obtained by simulated or measured transmission and reflection coefficients as shown in Figure S5b using Equation S1 and $(M_{0D})^{-1}$is given by

$$(M_{0D})^{-1} = M_{D0} = \frac{1}{2z_0}\begin{pmatrix} z_D + z_0 & z_0 - z_D \\ z_0 - z_D & z_D + z_0 \end{pmatrix} \quad \text{(S17)}$$

The transmission and reflection coefficients of air-MM-air configuration (Figure S5c), $t'_{12}, t'_{21}, r'_{12}$ and r'_{21}, can be calculated from M_{AMA} using Equation S1. In our work, we use $t'_{12}, t'_{21}, r'_{12}$ and r'_{21} to calculate χ_{se}, χ_{sm} (Equations S6 & S7), ε_{eff} and μ_{eff} (Equations S7-S11). To validate the method shown in equation S16, we simulated a 50 nm thick dielectric film with $\epsilon = 30$ and $\mu = 1$ on a BCB ($\epsilon_{BCB} = 2.3716, \mu_{BCB} = 1$) substrate as shown in Figure S5a. The transmission and reflection coefficients,t_{12}, t_{21}, r_{12} and r_{21}, for the air-MM-dielectric configuration are obtained directly from simulation within the wavelength range from 2μm to 10 μm. Meanwhile, t'_{12}, t'_{21}, r'_{12} and $r'_{21}$for the air-MM-air configuration is calculated using Equation 16. We calculated ε_{eff} and μ_{eff} using two sets of transmission and reflection coefficients as shown in Figure S5d,e. The air-MM-air configuration produces accurate values for both $\varepsilon_{eff} = 30$ and $\mu_{eff} = 1$ over the entire wavelength range from 2μm to 10 μm, while air-MM-dielectric configuration results in very large error and incorrect dispersion.

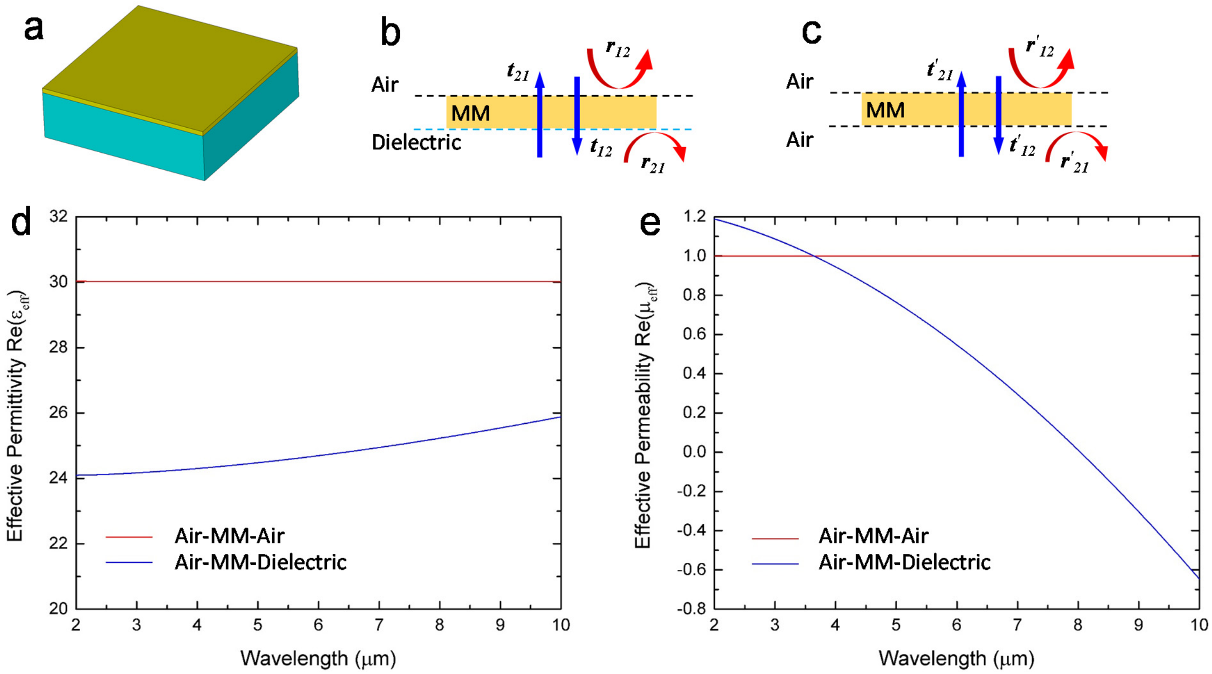

Figure S5. (a) Schematic model of a $d = 50\ nm$ thick dielectric film with $\epsilon = 30, \mu = 1$ on a 0.5 μm thick BCB layer. (b) The transmission and reflection coefficients, t_{12}, t_{21}, r_{12} and r_{21} at the air-film-dielectric (BCB) interfaces can be calculated in CST simulation using the model in (a). (c) $t'_{12}, t'_{21}, r'_{12}$ and r'_{21} at the interfaces of air-film-air are calculated by a transfer matrix method. (d),(e) The effective permittivity and permeability calculated using the transmission and reflection coefficients shown in (b) (blue curves) and (c) (red curves).

By modeling the MDA as a metafilm, the EM wave accumulate phase when the wave propagates through and reflected by the metafilm. This phase correctly represents the abrupt phase changes when the EM wave propagates through and reflects by the MDA. We perfomed full wave simualtion for air-MDA-spacer structure and the corresponding air-Metafilm-spacer structure, where the metafilm is defined by a 50nm-thick film with ε_{eff} and μ_{eff}. As shown in Fig. S6, the phases of transmission and reflecction coefficients of the metafilm perfectly match that of actual MDA structure.

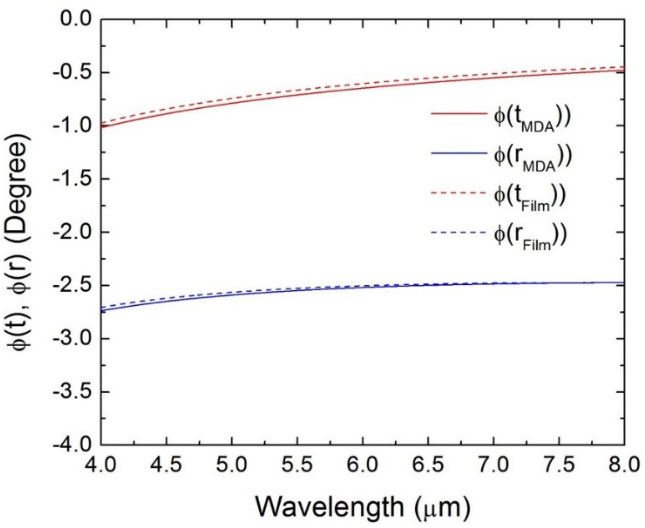

Figure S6. Simulated phase of transmission and reflection coefficients through MDA structure (solid) and metafilm (dashed).

References

1. Holloway CL, Dienstfrey A, Kuester EF, O'Hara JF, Azad AK, Taylor AJ. A discussion on the interpretation and characterization of metafilms/metasurfaces: The Two-Dimensional Equivalent of Metamaterials. *Metamaterials* 2009, **3:** 100.

2. Zhou J, Chen H-t, Koschny T, Azad AK, Taylor AJ, Soukoulis CM*, et al.* Application of metasurface description for multilayered metamaterials and an alternative theory for metamaterial perfect absorber. *arXiv:11110343v1* 2011.

3. Smith DR, Schultz S, Markos P, Soukoulis CM. Determination of effective permittivity and permeability of metamaterials from reflection and transmission coefficients. *Physical Review B* 2002, **65**(19)**:** 195104.
